# Macrophages Rapidly Seal off the Punctured Zebrafish Larval Brain through a Vital Honeycomb Network Structure

**DOI:** 10.3390/ijms231810551

**Published:** 2022-09-11

**Authors:** Dandan Zou, Jie Qin, Wenlong Hu, Zongfang Wei, Yandong Zhan, Yuepeng He, Congjian Zhao, Li Li

**Affiliations:** 1 Key Laboratory of Freshwater Fish Reproduction and Development, Ministry of Education, Institute of Developmental Biology and Regenerative Medicine, Southwest University, Beibei, Chongqing 400715, China; 2 Chongqing Engineering Research Center of Medical Electronics and Information Technology, School of Biomedical Engineering and Informatics, Chongqing University of Posts and Telecommunications, Chongqing 400065, China; 3 Research Center of Stem Cells and Ageing, Chongqing Institute of Green and Intelligent Technology, Chinese Academy of Sciences, Chongqing 400714, China

**Keywords:** macrophages, aggregation, stab, collagen, TBI

## Abstract

There is accumulating evidence that macrophages play additional important roles in tissue damage besides their typical phagocytosis. Although the aggregation of macrophages on injured sites has long been observed, few researchers have focused on the role of the overall structure of macrophage aggregation. In this study, we developed a standardized traumatic brain injury (TBI) model in zebrafish larvae to mimic edema and brain tissue spillage symptoms after severe brain trauma. Using time-lapse imaging, we showed that macrophages/microglia in zebrafish larvae responded rapidly and dominated the surface of injured tissue, forming a meaningful honeycomb network structure through their compact aggregation and connection. Disrupting this structure led to fatal edema-like symptoms with severe loss of brain tissue. Using the RNA-Seq, together with the manipulation of in vitro cell lines, we found that collagen IV was indispensable to the formation of honeycomb network structures. Our study thus revealed a novel perspective regarding macrophages forming a protective compact structure with collagen IV. This honeycomb network structure acted as a physical barrier to prevent tissue loss and maintain brain homeostasis after TBI. This study may provide new evidence of macrophages’ function for the rapid protection of brain tissue after brain injury.

## 1. Introduction

Zebrafish is a widely used model vertebrate [1,2]. Research on zebrafish is gradually extending to a variety of organs and systems [1,3], exemplified by the nervous system [4], immune system [5], cardiovascular system [6], reproductive systems [7], etc. The brain contains a wide spectrum of vital neural cells that are indispensable for an organism. Unfortunately, traumatic brain injury (TBI) caused by acupuncture or mechanical impact often disrupts the integrity and homeostasis of the brain, leading to serious and even fatal consequences [8,9]. TBI is a common form of brain injury with a high incidence and disability rate [10]. Studies in experimental models suggest that TBI can lead to neuroinflammation, brain edema and neuronal death [9,11]. Although there have been many reports of TBI models in adult zebrafish [12,13,14], the pathogenesis of cerebral edema after TBI still remains unclear. Therefore, it is necessary to conduct studies on the mechanism of cerebral edema after primary traumatic brain injury in zebrafish.

Central nervous system injury causes rapid responses of macrophages/microglia and surrounding immune cells such as neutrophils, monocytes and T cells [15]. As a major component of the innate immune system, macrophages are initially appreciated as canonical phagocytes that can phagocytose and digest pathogens and cell debris [16]. However, more and more research has revealed that, in addition to their typical phagocytic function, macrophages also play important roles in development, tissue remodeling, wound healing, angiogenesis and metabolism [17]. Macrophages are endowed with additional physical functions involving the secretion of trophic factors, especially when a tissue ruptures. For example, synovial macrophages are reported to build a dense physical barrier of membrane-like structures [18]. The resident tissue macrophages (RTM) are observed to cloak micro-lesions inside the body [19]. The primordial macrophages have been reported to serve as aggregated platelets to seal the damaged sites in the sterile injury of abdominal wall [20]. An investigation of the larval zebrafish muscle injury model revealed that macrophages can even provide a transient niche for muscle stem cells via producing NAMPT [21]. Apparently, in addition to being known as the pro- and anti-inflammatory executors of injury and repair, multiple recent studies have highlighted the novel roles of macrophages in the process of tissue restoration and regeneration. Meningeal macrophages are the first group of cells arriving at the injured site. These cells can lead to good prognosis due to the secretion of inflammatory factors such as IL-6 and TGF-β [22]. However, the complex cellular response, especially by the myeloid phagocytes of macrophages, to the brain injury at the initial acute stage remains poorly understood.

The extracellular matrix (ECM) is a scaffold that provides support for cell-cell interactions [23]. Adhesion between cells and ECM plays an important role in the migration, proliferation and differentiation of cells, especially during some physiological and pathological processes such as wound healing, inflammation and cancer metastasis [23,24]. Collagen and laminin serve as components of basement membranes, constituting a protective layer around capillaries and are included in the ECM to protect tissues from pathogens [25]. Collagen can maintain the structural integrity of tissues by interacting with a set of ECM components, including cell surface proteins and growth factors. Collagen type Ⅳ is the only type of collagen present in the basement membrane of vertebrates, differing from most other collagens [26,27]. Recently, the content of serum collagen type Ⅳ was considered as a reflection of collagen synthesis and was found to be positively correlated with the fibrosis process [28]. It is also considered to be critically involved in the process of tumor metastasis [29]. Previous studies have implied that collagen type Ⅰ can promote the aggregation of macrophages [30]. However, whether collagen type Ⅳ is related to the migration, recruitment and aggregated structure of macrophages after injury is an open question.

Here, we established a traumatic brain injury model in larval zebrafish and carefully analyzed the behavior of immune cells in the initial response after injury. We revealed that those macrophages/microglia immediately migrated and aggregated to the site of tissue injury. Interestingly, macrophages/microglia predominantly covered the surface of damaged brain tissue, forming a dense honeycomb network structure to prevent the outflow of brain tissue, which was critical for maintaining viable neuronal cells and efficient neuronal regeneration. The collagens, especially collagen type IV, played a crucial role in the aggregation of macrophages and structural integrity, which synergistically strengthened the closed connection to build a protective barrier.

## 2. Results

### 2.1. Macrophages Rapidly Aggregated to the Lesion Site to Form a Honeycomb Network Structure

**Figure 1:**
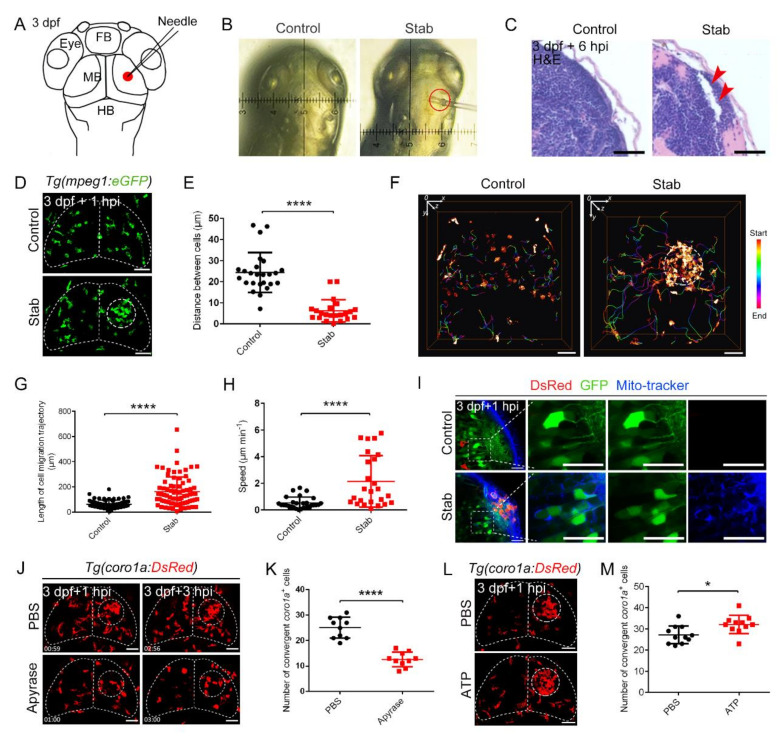
Predominant involvement of macrophages in response to traumatic brain injury caused by stabbing in zebrafish. (**A**) Stab model. The red dot marks the lesion core (LC). FB, forebrain; MB, midbrain; HB, hindbrain. (**B**) An actual operation diagram of the stab. The horizontal width of damage was about 130 μm. (**C**) A paraffin section was stained with hematoxylin and eosin (H&E) staining. The red arrowheads indicate the brain tissue loss. Scale bar, 20 µm. (**D**) The accumulation of macrophages in *Tg(mpeg1:GFP)* at 1 hpi. The white dotted line shows the major area of macrophage aggregation. Scale bar, 50 µm. (**E**) Statistical analysis of the distance between *coro1a*^+^ cells in LC. Control, 24.35 ± 1.89 µm *n* = 25; Stab, 6.14 ± 1.12 µm *n* = 22. (**F**) Spatial and temporal migration of macrophages. Scale bar, 50 µm. (**G**) Length statistics for macrophage migration trajectories. Control, 62.14 ± 3.881 µm, *n* = 71; Stab, 161.7 ± 13.42 µm, *n* = 78. (**H**) Statistical analysis of the movement speed of *coro1a*-DsRed^+^ cells after injury. Control, 0.5309 ± 0.08210 µm min^−1^ *n* = 28; Stab, 2.148 ± 0.3875 µm min^−1^ *n* = 25. (**I**) MitoTracker signals were detected in the damage sites. The right three panels are the enlarged views of the boxed regions in the left panel. Scale bar, 20 µm. (**J**) Aggregation of macrophages after apyrase injection in *Tg(coro1a:DsRed)* embryos at 1 hpi and 3 hpi. Scale bar, 50 µm. (**K**) Statistical number of *coro1a*^+^ cells after injury at 1 hpi. PBS, 25.10 ± 1.303 *n* = 10; apyrase, 12.60 ± 0.9092 *n* = 10. (L) Observation of macrophage aggregation after ATP injection at 1 hpi. Scale bar, 50 µm. (**M**) Statistical number of aggregated *coro1a*^+^ cells in (**L**). PBS, 27.18 ± 1.278 *n* = 11; ATP, 32.09 ± 1.317 *n* = 11. (Data are shown as mean ± SEM. *, *p* < 0.05; ****, *p* < 0.0001.)

Taking advantage of the optical transparency and regenerative capacity of larval zebrafish, we constructed a stable stab injury model in the left midbrain using a standardized needle at 3 dpf (Figure 1A,B), when microglial populations were established in the brain [31]. TdT-mediated dUTP nick-end labeling (TUNEL) and acridine orange (AO) staining assays were used to detect the apoptosis of brain tissue. The results indicate a drastic increase in TUNEL^+^ and AO^+^ signals on and around the wounds after injury (Figure S1A–C). High-resolution confocal imaging in a *Tg(HuC:eGFP)* transgenic reporter line [32] showed that a stab injury exerted a severe effect on neurons in the injured region, as evidenced by the significantly reduced fluorescence intensity of GFP^+^ cells (Figure S1D,E). Simultaneously, H&E staining of paraffin sections exhibited the loss of brain tissue at the injury sites (Figure 1C). These data suggested that our stab method effectively caused apoptosis and loss of neurons in zebrafish larvae.

Tissue damage promoted the accumulation and infiltration of various inflammatory cells, especially macrophages [33,34,35]. To investigate the dynamics of macrophages after brain injury, we performed confocal living imaging on a *Tg(mpeg1:eGFP)* transgenic line [36]. In this zebrafish line, *mpeg1^+^* macrophages were visualized by GFP signals. These GFP^+^ cells presented rapid migration and aggregation toward the wound site after injury (Figure 1D). The same phenomenon was also found in *Tg(coro1a:DsRed;mpeg1:GFP)* double transgenic lines [37] (Figure S2A). Through fluorescence co-localization analysis of DsRed^+^ and GFP^+^, we found that the number of *coro1a^+^mpeg1^+^* cells at the injury site increased significantly, accounting for 85.54% of the total aggregated *coro1a*^+^ cells (Figure S2B). This result indicates that most of *coro1a*^+^ cells aggregated in the damaged area were macrophages. Rapid movement of *mpeg1^+^* and *coro1a*^+^ cells allowed us to see significant cellular aggregation early after injury (Figure S2A,C; Movie S1). Statistically, they were tightly packed with an average membrane spacing of only 6.14 µm between adjacent *coro1a*^+^ cells, which was a quarter of the distance between closely packed cells under physiological control conditions (24.35 µm) (Figure 1E). These data suggest that macrophages responded rapidly in moving to the lesion site to form a dense structure.

### 2.2. The Recruitment of Macrophages to the Injury Sites Was Mediated by Nucleotide Messenger Molecules

We then focused on and analyzed the behavior of *coro1a*-Dsred^+^ cells. Under physiological conditions, the trajectories of *coro1a*^+^ cells in the optic tectum (OT) region were restricted to 62.14 ± 3.881 µm. In contrast, the active region of *coro1a*^+^ cells in the injured group expanded to an average of 161.7 ± 13.42 µm to move to the wound (Figure 1F,G, Movie S1). Statistically, the average velocity of active *coro1a*^+^ cells migrating to the wound was 2.15 ± 0.39 µm min^−1^, much higher than the velocity of spontaneous patrolling cells (0.53 ± 0.08 µm min^−1^) (Figure 1H).

It has been reported that high levels of adenosine triphosphate (ATP) can mediate rapid microglia responses to localized brain injury [38]. Therefore, we wanted to see if ATP levels increased at the center of the injury. MitoTracker, a specific mitochondrial marker with green fluorescence [39,40], showed a significant increase at the injured site (Figure 1I). Studies have shown that the “find me” signal that induced macrophages’ response was released from necrotic or apoptotic neurons, but not from living neurons or macrophages [41,42]. Our data showed that MitoTracker signals appeared around the damaged neurons, confirming previous studies showing that ATP was derived from damaged neurons [38]. To verify the induction of ATP in aggregated macrophages, we injected apyrase, a kind of ATP-hydrolase [38], into the lesion center and found that it effectively reduced the number of recruited *coro1a*^+^ and *mfap4^+^* cells at 1 hpi and 3 hpi (Figure 1J,K, Figure S2D–F). However, ATP supplementation significantly increased the number of gathered *coro1a*^+^ cells from 27.18 ± 1.278 to 32.09 ± 1.317 (Figure 1L,M). Pannexin-1 hemi-channels were preferentially expressed in zebrafish tectal neurons [43]. Previous research showed that knockdown of PANX1 had no effect on the apoptotic process, but affected the release of ATP and UTP from apoptotic cells, which were indispensable for the migration and recruitment of phagocytes [41,44]. Consistent with previous studies, the use of the pannexin-1 hemi-channel inhibitors probenecid (Pro) or carbenoxolone (CBX) significantly reduced the number of *coro1a*^+^ cells at the injury sites compared to the corresponding controls (Figure S2G,H). Collectively, the recruitment of *coro1a*^+^ cells to injury sites was mediated remotely by nucleotide messengers.

### 2.3. Recruited Macrophages in the Injured Sites Contained a Mixture of Localized Microglia and Adjacent Migrating Macrophages

To determine the source of ATP-induced *coro1a*^+^ cells that can accumulate to the lesion center immediately after injury, the cell proliferation of responding macrophages was considered. Edu (5-ethynyl-2′-deoxyuridine) is a thymidine nucleoside analogue that can be inserted into replicating DNA molecules during cell proliferation [45]. With combined use of Edu staining and dyes, cell proliferation analysis can be carried out efficiently and rapidly and cells in the S phase can be detected effectively [46]. Edu staining was used to detect the number of aggregated proliferating *coro1a*^+^ cells. However, Edu^+^ signals had no significant increase in injured larvae compared to non-aggregated control cells (Figure S3A,B).

**Figure 2:**
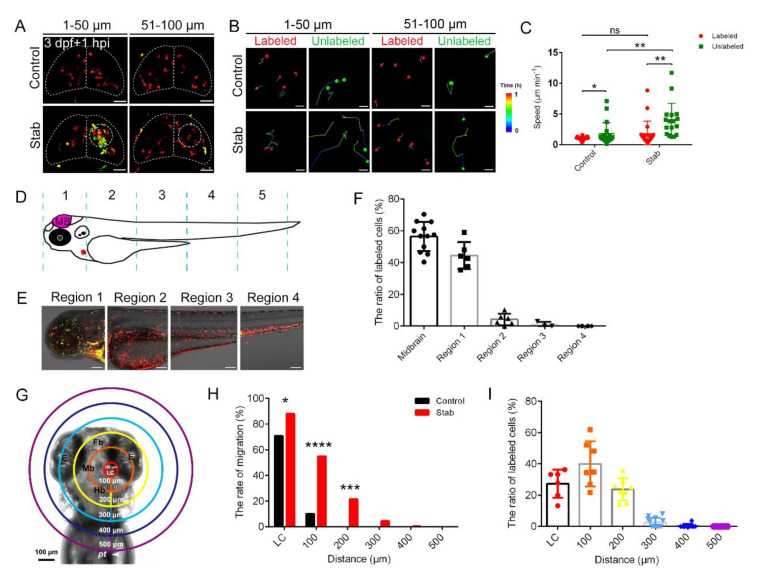
Macrophages around injured regions activated and responded in a distance-dependent manner. (**A**) The aggregated macrophages did not originate solely from the labeled *coro1a*-Kaede^+^ cells in the midbrain. Live imaging on the *coro1a*-Kaede^+^ cells in the upper (1–50 µm from surface, left panels) and deep (51–100 µm from surface, right panels) layers of *Tg(coro1a:Keade)* brains. Scale bar, 50 µm. (**B**) The migration trajectories of macrophages in different layers. Scale bar, 20 µm. (**C**) Statistical analysis of migration speed of labeled and unlabeled *coro1a*-Kaede^+^ cells. Control, labeled, 1.03 ± 0.05 µm min^−1^ *n* = 27; unlabeled, 1.79 ± 0. 42 µm min^−1^ *n* = 19; Stab, labeled,1.75 ± 0.46 µm min^−1^ *n* = 20; unlabeled, 3.95 ± 0. 68 µm min^−1^ *n* = 17. (**D**) Schematic diagram of regional division of zebrafish body. (**E**) *coro1a*^+^ cells in each region are labeled separately. Scale bar, 100 µm. (**F**) The proportion of labeled red *coro1a*-Kaede^+^ cells from different regions in aggregated cells. (**G**) Model diagram of region divisions at different distance from the LC. LC is a circle 100 microns in diameter at the lesion core. (**H**) The rate of migration of labeled cells in each region moving to the LC. (**I**) The contributing proportion of the cells from different regions to the accumulated macrophages of the honeycomb network configuration. (Data are shown as mean ± SEM. *ns*, no significance; *, *p* < 0.05; **, *p* < 0.01; ***, *p* < 0.001; ****, *p* < 0.0001.)

Microglia, the resident immune cells of the central nervous system, are usually the main cell type that initiates the inflammatory cascade when sensing danger of brain injury [47]. To elucidate the contribution of microglia, *Tg(coro1a:DsRed;apoe:GFP)* double transgenic lines were used. We found that *coro1a*^+^ cells at the injury sites contained not only *coro1a^+^ apoe^+^* microglia but also other *coro1a^+^ apoe^-^* macrophages (Figure S3C). To further explore contributing cells in the injured brain, we utilized the *Tg(coro1a:Kaede)* zebrafish line, in which the photoactivatable fluorescent protein Kaede labeled cells can change from green to red fluorescence upon 350–400 nm UV stimulation [48] to facilitate transient cell tracking. First, we labeled microglia in the midbrain with 405 nm UV light (Figure S3D). After clearly distinguishing red *coro1a*^+^ cells in the midbrain region from green *coro1a*^+^ cells in other regions, we performed stab experiments on labeled zebrafish. We found that red *coro1a*-Kaede^+^ cells contributed to approximately 56.37 ± 2.66% of aggregated cells at 1 hpi (Figure 2A). Remarkably, green *coro1a*^+^ cells, which were located outside the midbrain and needed to travel a longer distance to the injured site, were able to migrate faster to reach the center of the lesion in time (Figure 2B,C). In contrast, green *coro1a*-Kaede^+^ cells rarely moved to the midbrain under physiological conditions (Figure 2A).

Next, we wanted to know how far the aggregated *coro1a*^+^ cells can migrate before reaching their destination. We partitioned the zebrafish body into six parts. The midbrain was designated as the MB region and the non-MB region of the zebrafish head position was defined as region 1. From the yolk sac to the tail, each 500 µm distance was designated as an individual region, such as region 2 to region 5 (Figure 2D). To investigate the proportion of macrophages migrating to the lesion center in each region, macrophages of each region were labeled as red *coro1a*^+^ cells (Figure 2E). At 1 hpi, we counted the proportion of red *coro1a*^+^ cells migrated to the center of injury. We found that aggregated macrophages were mainly from the MB region and region 1. Only 4.175 ± 1.472% of the aggregated cells were from region 2 (Figure 2F). These results imply that macrophages responded in a distance-dependent manner after injury, which warranted careful estimation. Hence, we defined a circle of 100 µm centered at the injured site as the criterion for the lesion center (LC). Subsequently, macrophages located in regions of different distance from the LC were labeled orderly and their migration and contribution to LC aggregated cells were meticulously assessed (Figure 2G). Data analysis revealed that 87.94 ± 3.91% of *coro1a*-Kaede^+^ cells originally located in the LC remained in the same region after injury, representing 27.36 ± 3.67% of the total aggregated cells. Overall, 54.92 ± 2.10% of labeled *coro1a*-Kaede^+^ cells within 100 μm from LC migrated to the LC, accounting for 40.01 ± 5.47% of the total aggregated cells. In the region of 100–200 µm from the LC, 21.59 ± 2.40% of labeled *coro1a*-Kaede^+^ cells migrated to the LC after injury, accounting for 23.68 ± 2.54% of the total aggregated cells. The number of aggregated cells from the region 200–300 µm from the LC was very limited (only about 2.63 ± 0.87%) and there were few cells further than 300 µm from the LC that could respond in time to TBI (Figure 2H,I and Figure S3E). These results imply that *coro1a*-Kaede^+^ cells within 200 µm from the center of injury activated in a distance-dependent manner soon after TBI.

### 2.4. Accumulation of Sufficient Macrophages Was Required for the Establishment of Honeycomb Network Structures on the Surface of Injured Brain

**Figure 3:**
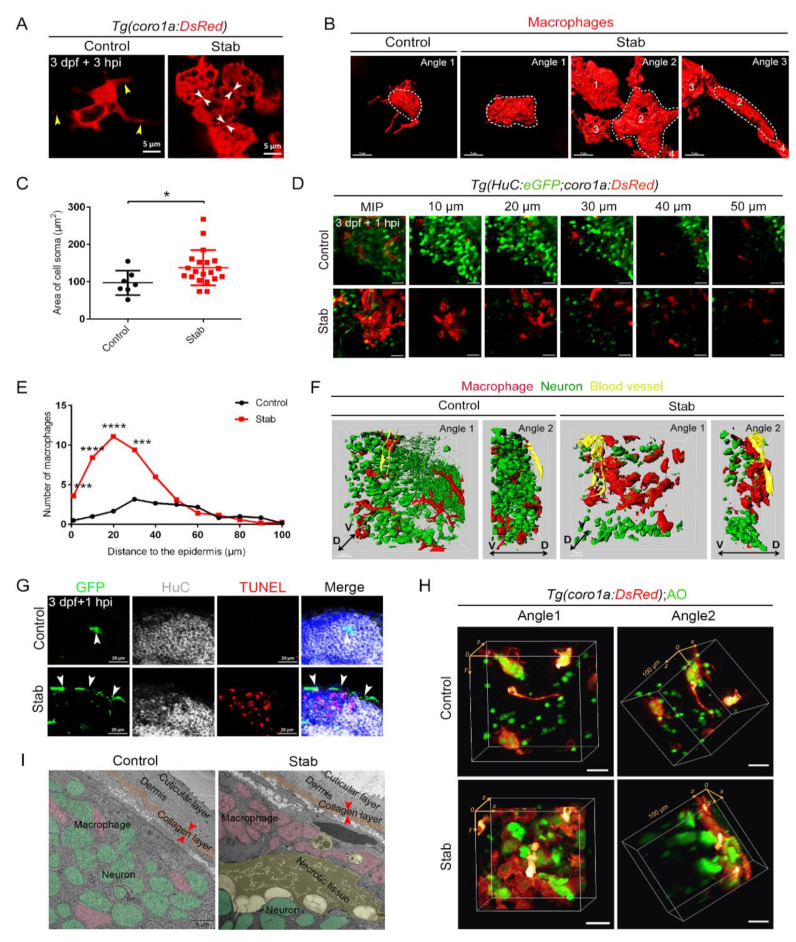
Aggregated macrophages formed a honeycomb network structure covering the wound surface. (**A**) Changes in macrophages morphology after injury. The yellow arrowheads indicate the branches. The white arrowheads indicate tight connections between cells. Scale bar, 5 µm. (**B**) Three-dimensional reconstruction of the morphology of macrophages after injury. Angle 1 shows the morphology of individual cells and Angles 2 and 3 indicate tight connections between cells. Scale bar, 7 µm. (**C**) Statistic analysis of the area of cell soma surface at 3 hpi. Control, 97.08 ± 12.37 µm^2^ *n* = 7; Stab, 137.50 ± 10.61 µm^2^ *n* = 20. (**D**) Distribution of macrophages and neurons on different layers of the Z axis at 1 hpi. The number on each panel represents the distances to the surface. Scale bar, 20 µm. (**E**) Statistics analysis of *coro1a*^+^ cells number at different layers with distinct distances from the epidermis at 1 hpi. (**F**) Three-dimensional images of the distribution of macrophages (red) and neuron (green) in *Tg(coro1a:DsRed;HuC:eGFP)* at 1 hpi. Yellow marks the blood vessels. D, dorsal; V, ventral. Scale bar, 15 µm. (**G**) The immunofluorescent images of GFP, HuC and TUNEL on the frozen sections of *Tg(coro1a:eGFP)* brains at 1 hpi. The white arrowheads present the distribution of GFP^+^ cells. Scale bar, 20 µm. (**H**) The location of macrophages and AO^+^ signals in 3D view. Scale bar, 20 µm. (**I**) Transmission electron microscope image. Macrophages are in pink, neurons are in green, apoptotic or necrotic tissue is in yellow and the collagen layer beneath the skin is in orange. The red arrowheads indicate the width of the collagen layer. Scale bar, 5 µm. (Data are shown as mean ± SEM. *, *p* < 0.05; ***, *p* < 0.001; ****, *p* < 0.0001.)

**Figure 4:**
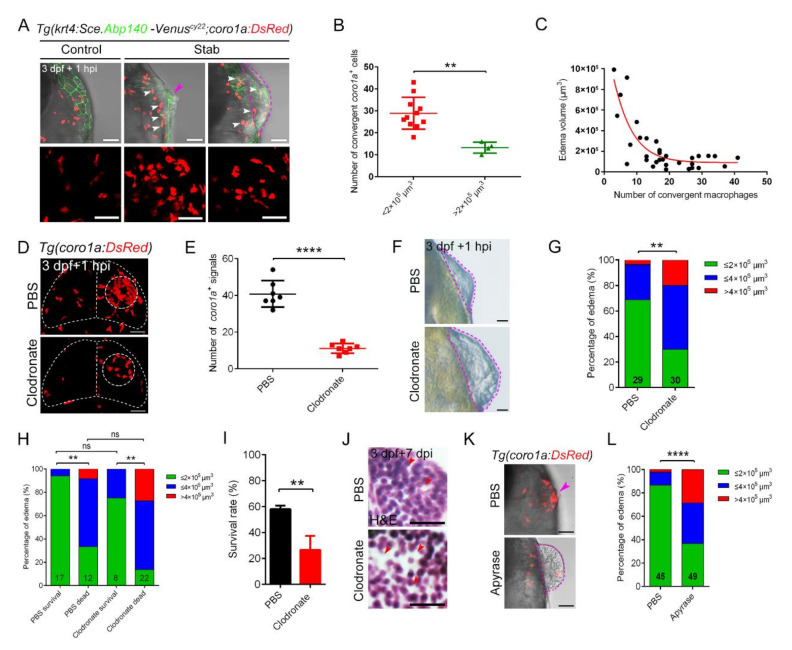
The dense structure of macrophages played an important role in maintaining brain integrity and survival of injured zebrafish larvae. (**A**) Edematous phenotypes appeared in injured *Tg(coro1a:DsRed;krt4:Sce.Abp140-Venus)^cy22^* with a few macrophage aggregations. The white arrowheads in upper images indicate the distribution of macrophages after injury. Purple arrowhead indicates a mild edematous phenotype. The purple dotted line indicates a severe edematous phenotype. Scale bar, 50 µm. (**B**) Statistical analysis of aggregated *coro1a*^+^ cells number in (**A**). Control, 28.91 ± 2.19 *n* = 11; Stab, 13.25 ± 1.25 *n* = 4. (**C**) The correlation between edema-like symptoms and the number of aggregated macrophages. (**D**) The distribution of macrophages after injury with clodronate liposomes or PBS injection. Scale bar, 50 µm. (**E**) Statistical analysis of aggregated *coro1a*^+^ cells in (**D**). PBS, 40.86 ± 2.72 *n* = 7; Clodronate, 11.14 ± 0.99 *n* = 7. (**F**) Observation of edema-like symptoms after injury. Scale bar, 50 µm. (**G**) Statistics of edematous symptoms in (**F**). (**H**) Analysis of zebrafish survival and edematous symptoms in Clodronate/PBS treatment group after injury. (**I**) Survival rate of zebrafish at 1 dpi. (**J**) H&E staining of brain tissue at 7 days after injury. Scale bar, 10 µm. Red arrowheads indicate locations of apparent tissue loss. (**K**) The bright field and fluorescent images of *coro1a*-DsRed^+^ cells and edema-like symptoms by treatment with Apyrase/PBS. Scale bar, 50 µm. Purple arrowhead indicates a mild edematous phenotype. The purple dotted line indicates a severe edematous phenotype. (**L**) Statistics of edematous symptoms by treatment with apyrase/PBS. (Data are shown as mean ± SEM. *ns*, no significance; **, *p* < 0.01; ****, *p* < 0.0001.)

It is worth noting that damage-induced aggregated *coro1a*-DsRed^+^ cells were morphologically vacuolated with significantly reduced branching (Figure 3A and Movie S2). The 3D images of macrophages clearly showed that, after injury, the cell body of macrophages appeared to be more stretched, with a significantly enlarged cross-section area and an oblate cell morphology (Figure 3B,C). Furthermore, the aggregated macrophages were closely clustered, overlapped and intercrossed to form a typical honeycomb network structure (Figure 3A,B). The obvious aggregation of *coro1a*-Kaede^+^ cells at the wound surface (Figure 2A) led us to investigate whether a similar phenomenon recurred in the deep brain parenchyma. The injured area of approximately 100 µm from the wound surface to the wound depth was examined in *Tg(coro1a:DsRed;HuC:eGFP)* larvae. Surprisingly, a limited number of *coro1a*^+^ cells were detected in the deep layers, but a considerable number of cells were observed less than 30 µm from the wound surface. In contrast, *coro1a*^+^ cells in the control group, which were regularly scattered inside the brain parenchyma, were barely visible on the surface (Figure 3D–F and Movie S3). Careful observation indicated that the *coro1a*-DsRed^+^ cells converged to form a dense structure on the damaged surface (Figure 3F). These cells were not present in the damaged tissue within the brain where neurons showed apparent death (Figure 3G,H and Movie S4). The intriguing migration and aggregation characteristics of macrophages were further validated by transmission electron microscopy (TEM). Macrophages presenting stromal bodies were tightly packed beneath the collagen layer of the injured brain. They were in close contact with each other, while some of them engulfed the apoptotic cells (Figure 3I). However, these cells did not appear in the regions of necrotic tissue in close vicinity (Figure 3I). Together, these data indicate the predominant involvement of macrophages through the rapid construction of honeycomb network structures in response to brain injury, which probably served as a fortress against parenchymal outflow. Serendipitously, we found that a small population of stabbed larvae (about 6%) failed to achieve the aggregation of macrophages due to the dispersed distribution and compromised interaction of *coro1a*-DsRed^+^ cells at the injury sites. Statistically, the number of recruited *coro1a*^+^ cells in these larvae was approximately 13.25 ± 1.25. This was half that its counterpart (28.91 ± 2.189), concentrated in the dense honeycomb structures (Figure 4A,B). Intriguingly, a massive outflow of brain tissue was immediately observed at 1 hpi, forming edema-like structures with a volume exceeding 2 × 10^5^ µm^3^ (Figure 4A). Measurements of macrophage number and edema volume revealed an interesting correlation. When the number of macrophages accumulated at the injury site was less than 15, notable edema-like symptoms could be observed after TBI (Figure 4C). This phenomenon inspired us to explore the requirement regarding the number of macrophages for the construction of honeycomb structures (Figure 4C). To this end, we injected larval zebrafish with clodronate liposomes for the specific killing of macrophages [49,50]. Fourteen hours after the injection of clodronate liposomes, macrophages died significantly, accompanied by the reduction in the number of *coro1a*^+^ cells and loss of *apoeb^+^* signals (Figure S4A). Accordingly, the number of converging cells at the injury site was reduced to 11.14 ± 0.99, which was approximately a quarter of the number of aggregated cells in the control groups (40.86 ± 2.72) (Figure 4D,E). As a result, more severe edema was observed in the clodronate liposomes injected group than the contrast (Figure 4F). A further chi-square test indicated that the presence of the edema-like phenotypes after clodronate liposomes injection was associated with macrophages depletion (Figure 4G). The appearance of edema-like symptoms led to a higher mortality rate in zebrafish after injury (Figure 4H). In addition, surviving zebrafish larvae exhibited extensive loss of neuronal tissue even after 7 days, which was much more severe than control groups that undergone better restoration and regeneration of brain tissue (Figure 4I). Meanwhile, enlarged edema and increased lethality similarly occurred in the larvae after application of apyrase, probenecid (Pro) and carbenoxolone (CBX) (Figure 4J,K and Figure S4B,C), which presented incomplete macrophage convergence at the injury sites (Figure S2D–H). Together, these data indicated that sufficient macrophages were required to build the protective structures that maintained the integrity of brain tissue after injury.

### 2.5. Aggregated Macrophages Were Critical for Preventing Outflow of Neuronal Cells

**Figure 5:**
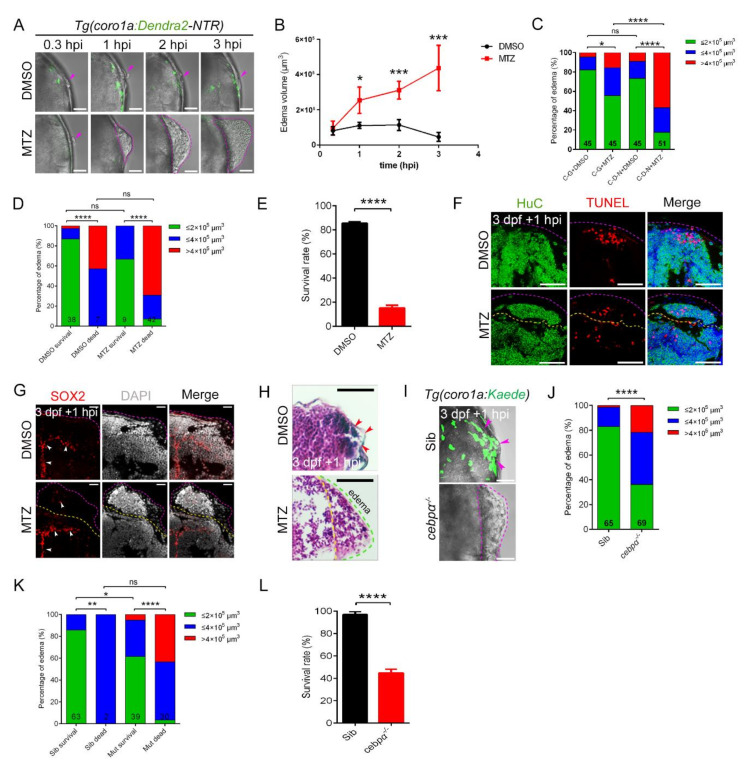
The absence of macrophages led to the development of brain edema. (**A**) Phenotypes of edema-like symptoms at different time points after injury. Scale bar, 50 µm. (**B**) Statistics of edema volume in (**A**). (**C**) Statistics of edematous symptoms in *Tg(coro1a:Dendra2—NTR)* (C-D-N) and *Tg(coro1a:GFP)* (**C**–**G**) with DMSO/MTZ treatment. Purple arrowheads indicate the injury sites. (**D**) Analysis of zebrafish survival and edematous symptoms in *Tg(coro1a:Dendra2-NTR)* with DMSO/MTZ treatment after injury. (**E**) Survival rate of *Tg(coro1a:Dendra2-NTR)* with DMSO/MTZ treatment at 1 dpi. (**F**) The fluorescent images of HuC^+^ and TUNEL^+^ signals in edematous overflow. The purple dotted line represents the lateral edge of the edema and the yellow dotted line represents the internal measurement edge of the edema. Scale bar, 50 µm. (**G**) The fluorescent images of SOX2^+^ signals in edematous overflow. The red arrowheads indicate location of SOX2^+^ signals. Scale bar, 20 µm. (**H**) Detection of brain tissue by H&E staining of paraffin sections after injury. Red arrowheads indicate the injury site. Scale bar, 50 µm. (**I**) The bright field and fluorescent images of *coro1a*-Kaede^+^ cells and edema-like symptoms (purple dashed line). Scale bar, 50 µm. The purple arrowheads indicate the accumulation of macrophages at the wound site. (**J**) Statistics of edematous symptoms in *cebpα* mutant and sibling after injury. (**K**) Analysis of zebrafish survival and edematous symptoms in *cebpα* mutant and sibling after injury. (**L**) Survival rate of *cebpα* mutant and sibling at 1 dpi. (Data are shown as mean ± SEM. *ns*, no significance; *, *p* < 0.05; **, *p* < 0.01; ***, *p* < 0.001; ****, *p* < 0.0001.)

The response of macrophages to aggregation after injury motivated us to explore the outcomes when macrophages were completely interfered. We ablated macrophages by treating the transgenic lines *Tg(coro1a:Dendra2-NTR)* with metronidazole (MTZ). Bacterial nitro-reductase (NTR) allowed the transient deletion of *coro1a*^+^ cells after treatment of MTZ according to the specific damage principle of MTZ/NTR system. NTR was reduced by NADH or NADPH, transforming non-toxic MTZ into cytotoxic metabolites, which specifically led to the death of NTR-expressing *coro1a*^+^ cells [51] (Figure S5A). Our data showed that macrophages were significantly reduced in *Tg(coro1a:Dendra2-NTR)* zebrafish whole embryos after MTZ treatment compared to controls, especially in the head (Figure S5B,C). After ablation of macrophages, there was no *coro1a*^+^ cells aggregation at the injury sites. We observed notable edema-like symptoms at the injury sites (Figure 5A). We found that the volume of edema in the DMSO group peaked at 1 hpi and then the symptoms of edema gradually decreased until they disappeared. However, the edema volume in the macrophage-depleted MTZ group was significantly higher than that in the DMSO group at 1 hpi and even expanded in the subsequent 2 h (Figure 5A,B). Notable outflow of the injured tissue and severe edema-like phenotypes with volume exceeding 2 × 10^5^ µm^3^ were observed in approximately 82.35% of MTZ-treated samples, while limited protrusion was observed in DMSO-treated samples (Figure 5C). We counted and grouped edema samples of different volume and performed the chi-square test for edema-like symptoms in DMSO and MTZ group. The results show that edema symptoms closely related to macrophage loss (Figure 5C). Further analysis showed that zebrafish with severe edema-like symptoms in the MTZ-treated *Tg(coro1a:Dendra2-NTR)* group had a lower survival rates (Figure 5D). Sections of the overflow tissue showed that there were a large number of live HuC^+^ neurons and a small number of SOX2^+^ neural precursor cells, which were not co-stained with TUNEL^+^ signals (Figure 5E–G). This suggested that the severe loss of viable neurons due to edema-like symptoms contributed to the death of the zebrafish. To estimate the potential toxicity of MTZ, we conducted the same experiment in *Tg(coro1a:eGFP)* [37], in which macrophages were not significantly affected by MTZ treatment (Figure S5C). The results show that there was no significant difference between *Tg(coro1a:Dendra2-NTR)* and *Tg(coro1a:GFP)* treated with DMSO, but high concentrations of MTZ also induced a fraction of edema-like symptoms after brain injury in *Tg(coro1a:eGFP)* zebrafish (Figure 5C). To rule out the possible effects of MTZ on edema-like symptoms, we obtained a *cebp*α mutant completely devoid of myeloid phagocytes (Figure S5D–G). *Coro1a*^+^ and *apoeb^+^* cells were not detected in the mutant, similar to those reported previously [52] (Figure S5H). After injury, there was no macrophage aggregation in mutant zebrafish larvae (Figure 5H). In addition, up to 60% of *cebpα* mutant larvae showed edema-like phenotypes after injury, compared to less than 20% in the wild-type (Figure 5I). The survival rate of siblings was statistically as high as 96.9%, while mutants had significantly reduced survival after injury. After statistical analysis, severe mortality in mutants after injury was also associated with the appearance of edema-like symptoms (Figure 5J). In addition, we excluded platelet differences between sibling and mutant larvae (Figure S5I), indicating that the edema-like phenotypes were not due to insufficient coagulation. Collectively, we hypothesized that the recruitment of macrophages to generate a condensed honeycomb network structure played an important role in preventing the outflow of parenchyma, which was essential for larval survival after injury.

### 2.6. Phagocytosis of Aggregated Macrophages Had Little Effect on the Protection of Brain Tissue after Injury

**Figure 6:**
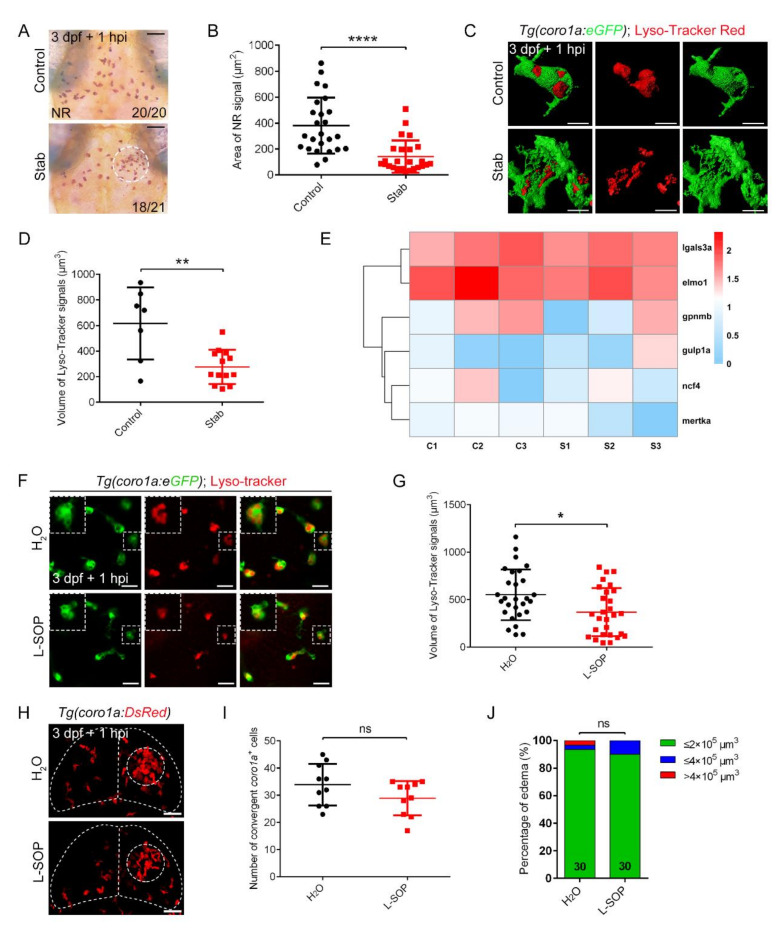
Phagocytosis played limited roles in controlling edematous symptoms. (**A**) Neutral red staining at 1 hpi. Scale bar, 50 µm. (**B**) Area statistics of neutral red signals in (**A**). Control, 381.2 ± 43.43 µm^2^ *n* = 25; Stab, 141.8 ± 24.82 µm^2^ *n* = 25. (**C**) The fluorescent images of *coro1a*-GFP^+^ and Lyso-Tracker Red^+^ at 1 hpi. Scale bar, 10 µm. (**D**) Statistical volume of Lyso-Tracker Red^+^ signals in (**C**). Control, 615.60 ± 106.50 µm^3^, *n* = 7; Stab, 276.30 ± 36.79 µm^3^, *n* = 13. (**E**) Heat map of phagocytic genes expression. C, control; S, stab. (**F**) The fluorescent images of *coro1a*-GFP^+^ and Lyso-Tracker Red^+^ after injecting O-phospho-L-serine (L-SOP)/ /H_2_O. Scale bar, 20 µm. (**G**) Statistical volume of Lyso-Tracker Red^+^ signals in (**F**). H_2_O, 551.4 ± 50.54 µm^3^ *n* = 28; L-SOP, 367.8 ± 47.79 µm^3^ *n* = 28. (**H**) Observation of macrophage aggregation after injecting L-SOP/H_2_O. Scale bar, 50 µm. (**I**) Statistical analysis of *coro1a*^+^ cells aggregated in (**H**). H_2_O, 33.90 ± 2.406 *n* = 10; L-SOP, 28.90 ± 1.997 *n* = 10. (**J**) Statistics of edematous symptoms after the application of L-SOP/H_2_O. (Data are shown as mean ± SEM. *ns*, no significance; *, *p* < 0.05; **, *p* < 0.01; ****, *p* < 0.0001).

Herzog’s study suggested that microglia played an important neuroprotective role after brain injury by phagocytosing cell debris [53]. Although our study suggested that microglia represent only a subset of aggregated cells, we wondered whether aggregated macrophages/microglia play a protective role in brain injury through phagocytosis. We first investigated the possible effect of phagocytosis by performing neutral red (NR) staining, which is commonly used to evaluate the phagocytosis of macrophages, by analyzing the uptake of neutral red in zebrafish [54,55,56]. In our study, despite the remarkable increase in the number of NR^+^ cells at the stab wound sites, the area of NR^+^ signals was much smaller than that of control at 1 hpi (Figure 6A,B). The same results were confirmed with Lyso-Tracker Red, a fluorescence probe that selectively accumulated in low-internal pH cellular compartments such as lysosomes [57]. Lyso-Tracker Red was commonly used as a lysosomal marker to test the integrity [58] and activity [59] of lysosomes. Although aggregated cells were vacuolated (Figure 3A and Figure 6C), their lysosomal volume was significantly reduced (Figure 6C,D). These data implicated a limited role of phagocytosis of aggregated macrophages. These findings are also consistent with the limited distribution of macrophages at deeper sites of injury, where many apoptotic signals appeared (Figure 3G–I). To elucidate the mechanisms behind the protective role of aggregated macrophages post-injury, we labeled and isolated aggregated red *coro1a*-Kaede^+^ cells in the LC and their uninjured controls from the same location for RNA sequencing (Figure S6A). Subsequent transcriptome analysis identified 401 differentially expressed genes, of which 230 and 171 genes were transcriptionally up- and down-regulated, respectively (Figure S6B). However, phagocytosis and/or trophic factor-associated signaling pathways, such as Fc gamma R-mediated phagocytosis, did not manifest significant alterations (ko04666). Typical phagocytosis-associated genes (GO:0006909), such as *lgals3a* [60] and *elmo1* [61], were not significantly changed (Figure 6E). Accordingly, these transcriptome readouts provided a reference for limited phagocytosis of macrophages from the gene expression levels. In order to further verify the effect of macrophages phagocytosis on edema-like symptoms, we used O-phospho-L-serine (L-SOP), a drug that can inhibit the phagocytic ability of microglia [53], to affect the phagocytosis of macrophages. Consistently, after L-SOP treatment, the phagocytic capacity of macrophages was significantly impacted, as manifested by a significant decrease in the volume of Lyso-Tracker Red^+^ signal (Figure 6F,G). However, it had less effect on the recruitment and aggregation of *coro1a*-DsRed^+^ cells (Figure 6H,I). There was no statistically significant difference in the proportion of post-injury edema-like phenotypes between L-SOP-treated and PBS-treated groups. It is worth noting that the treatment of L-SOP only inhibited macrophage phagocytosis without affecting their aggregation ability (Figure 6J). These results suggest that phagocytosis played a limited role in the protective function of aggregated macrophages.

### 2.7. Collagen Played a Crucial Role in the Construction of Honeycomb Network Structures

**Figure 7:**
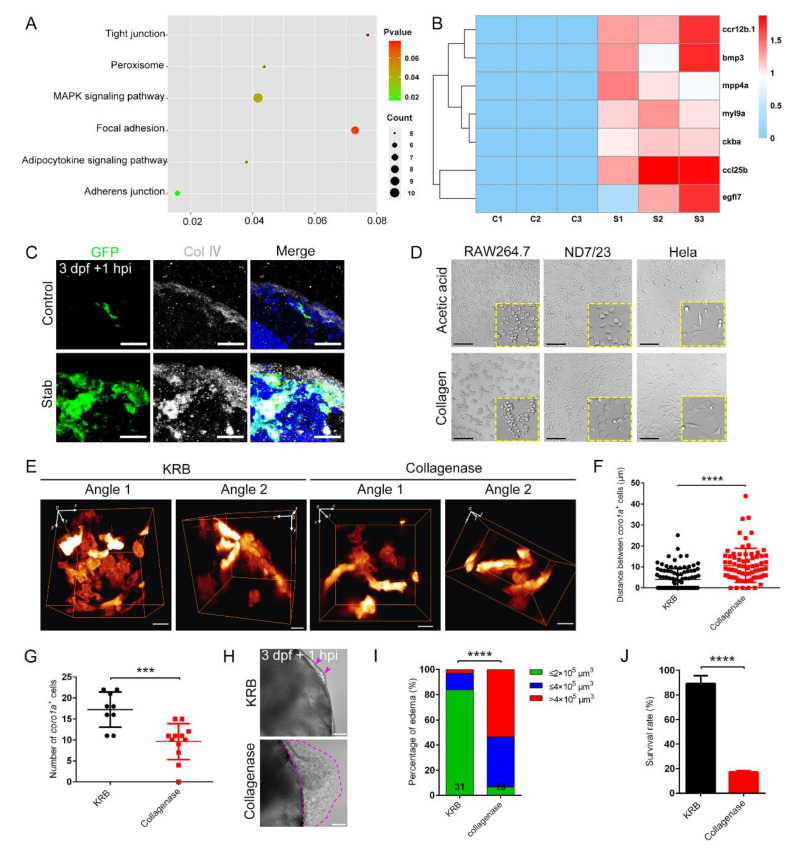
Collagenase disrupted macrophage aggregation and caused severe edematous symptoms. (**A**) Analysis of the altered signaling pathways in labeled red *coro1a*-Kaede^+^ cells before and after injury. (**B**) Heat map of changed representative genes. (**C**) Immunofluorescence imaging of GFP and type Ⅳ collagen (Col Ⅳ) on frozen sections of stabbed *Tg(coro1a:eGFP)* brain. Scale bar, 20 µm. (**D**) Observation of cell aggregation in the RAW264.7 cell lines, HeLa cell lines and ND7/23 cell lines with collagen/acetic acid added. Scale bar, 100 µm. (**E**) The distribution of macrophages after collagenase treatment in *Tg(coro1a:DsRed)* embryos at 1 hpi. KRB, Krebs–Ringer bicarbonate buffer, collagenase solvent. Scale bar, 10 µm. (**F**) Statistical analysis of distances between the macrophages in (**E**). KRB, 4.01 ± 0.53 µm *n* = 96; Collagenase, 10.67 ± 0.94 µm *n* = 75. (**G**) Statistical analysis of aggregated *coro1a*^+^ cells number in (**E**). KRB, 17.22 ± 1.40 *n* = 9; Collagenase, 9.58 ± 1.23 *n* = 12. (**H**) Observation of edematous symptoms (purple dished lines) after collagenase/KRB injection. Purple arrowheads indicate a mild edematous phenotype. Scale bar, 50 µm. (**I**) Statistics of edematous symptoms of the injured larvae after collagenase/KRB injection. (**J**) Survival rate of the stabbed larvae after collagenase/KRB injection. (Data are shown as mean ± SEM. ***, *p* < 0.001; ****, *p* < 0.0001.)

RNA sequencing analysis revealed that the mitogen-activated protein kinase (MAPK) signaling pathway, which is involved in various cellular functions including cell proliferation, differentiation and migration, was altered after injury (Figure 7A). Meanwhile, the expression of *ccl25b* and *ccr12b.1* involved in chemotaxis (GO:0006935) was dramatically increased in the injured group compared to the uninjured group. These data are consistent with previous observations of rapid migration of macrophages (Figure 1F,H). Furthermore, Kyoto Encyclopedia of Genes and Genomes (KEGG) analysis revealed that altered factors were highly involved in cell junction related pathways, including tight junctions, focal adhesions and adherens junctions (Figure 7A). Similarly, the heat map data unveiled the upregulation of focal adhesion (ko04510)- and tight junction (ko04530)-associated genes (*mpp4a*, *myl9a* and *myl9b*) and other genes (*bmp3*, *egfl7* and *ckba*) related to the extracellular region (GO:0005576) and cell surface (GO:0009986) after injury (Figure 7B). These data are consistent with the impressive appearance of honeycomb network structures to protect tissue integrity after TBI (Figure 3A).

At the same time, we wanted to explore whether adhesive proteins were involved in the existence of the tight aggregation of macrophages. Several canonical adhesion proteins, including collagen, laminin and integrin, were assessed by the immunofluorescence staining at the injured and uninjured sites. The results indicate that these proteins significantly increased at the injury sites compared to the corresponding uninjured control sites. Moreover, *coro1a*^+^macrophages co-localized with the collagen IV^+^, laminin^+^ and integrin^+^ layers at the injury sites (Figure 7C and Figure S6C,D). These findings suggest that adhesion proteins are plausibly responsible for the tight alignment and construction of honeycomb network structures by aggregated macrophages to protect the injured sites and facilitate tissue regeneration. We further corroborated our findings by examining the effects of collagen IV and laminin in different cell lines in vitro. Our results show that both factors effectively induced convergence and tight alignment in the macrophage cell line RAW264.7, but not in other cells such as dorsal root ganglions (neuroblasts, ND7/23) and HeLa (Figure 7D and Figure S6E). Meanwhile, other factors, such as bovine serum albumin (BSA), were not effective in inducing the aggregation of RAW264.7, similar to the phenotype of cells cultured with PBS as a control (Figure S6F). In addition, the denaturation of the collagen by heating resulted in the drastic disappearance of aggregated RAW264.7 cells (Figure S6E). These results suggest the important role for collagen and/or laminin in macrophage aggregation and adhesion.

To confirm the critical function of collagen in macrophage aggregation, we applied collagenase, which effectively disrupted the structure of collagen, to cultured cells, resulting in the reduced aggregation of macrophages (Figure S6E). Likewise, when additional collagenase was injected at the site of injury in vivo, the *coro1a*^+^ cells were scattered and irregularly arranged and their spacing increased 2.5-fold compared to controls (Figure 7E,F). At the same time, the number of *coro1a*^+^ cells accumulated at the injured sites decreased to 9.58 ± 1.23 (Figure 7G). In addition to significantly increasing the lethality of injured larvae compared to untreated controls, it also resulted in increased edema-like symptoms (Figure 7H,J). These data reminded us that collagen can help macrophages to clump together, thereby inhibiting tissue loss and the development of edema-like symptoms.

### 2.8. The Cooperation of Aggregated Macrophages and Collagen Was Essential for the Functional Protection of Honeycomb Network Structures

**Figure 8:**
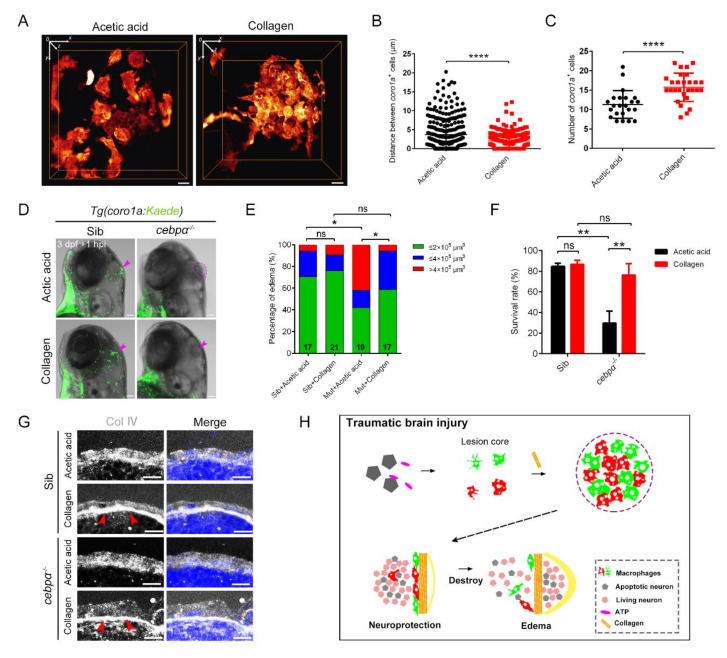
Collagen was crucially involved in the construction of the honeycomb network structure and the protection of brain integrity. (**A**) The distribution of macrophages after collagen/acetic acid injection in *Tg(coro1a:DsRed)* embryos at 1 hpi. Scale bar, 10 µm. (**B**) Statistical analysis of distances between the *coro1a*-DsRed^+^ cells in (**A**). Acetic acid, 3.73 ± 0.21 µm *n* = 340; collagen, 2.05 ± 0.10 µm *n* = 351. Acetic acid, collagen solvent. (**C**) Statistical number of aggregated *coro1a*^+^ cells in (**A**). Acetic acid, 11.30 ± 0.75 *n* = 23; collagen, 15.75 ± 0.69 *n* = 28. (**D**) The bright field and fluorescent images of *coro1a*-Kaede^+^ cells and edema-like symptoms (purple arrowheads and dashed line) in sibling and *cebpα* mutant with collagen/acetic acid injection. Scale bar, 100 µm. (**E**,**F**) Statistics of edematous symptoms (**E**) and survival rate (**F**) after collagen/acetic acid injection in *cebpα* mutant and sibling. (**G**) The immunofluorescence staining of collagen (Col Ⅳ) in *cebpα* mutant after injection of collagen. Scale bar, 10 µm. Red arrowheads indicate dense collagen layers. (**H**) Diagram of our results. The rapid and efficient construction of the honeycomb network structure by aggregated macrophages/microglia required nucleotide-mediated migration and collagen supportive tight junctions. The honeycomb network structures played an important role in the integrity of the damaged brain to protect the brain components inside. (Data are shown as mean ± SEM. *ns*, no significance; *, *p* < 0.05; **, *p* < 0.01; ****, *p* < 0.0001.)

Next, we wanted to see if additional injections of collagen could rescue edema-like symptoms. Type Ⅳ collagen was the only member of the collagen superfamily that can form molecular networks to induce cell adhesion, migration and differentiation [26]. Injection of collagen IV into the injury sites resulted in the augmented and intensive aggregation of *coro1a*-DsRed^+^ cells, exhibiting enhanced tight aggregation with reduced intervals (1.85-fold) at the wound sites (Figure 8A–C). These phenotypes implied the impressive ability of collagen to facilitate macrophages attachment and adhesion to build honeycomb network structures. Considering the TEM images of regular distribution of macrophages (Figure 3I) and their physical adhesion to the collagen layer, the crucial role of this mechanically tight “concrete” of macrophages and collagen in rapidly sealing injury sites cannot be ruled out. Therefore, enhancing the collagen layer should affect the formation of protective honeycomb network structures by macrophages after injury. To validate this hypothesis, we assessed the effect of collagen Ⅳ supplementation in *cebpα* mutant. Interestingly, the significant improvement of edema-like symptoms and survival rate in injured larvae was observed despite the loss of macrophages (Figure 8D–F). Furthermore, a dense layer of collagen IV was simultaneously detected under the skin (Figure 8G). However, the extra injection of laminin in *cebpα* mutant did not alleviate the edema-like symptoms, although laminin increased after injury and its addition to the cultured cells induced macrophage aggregation (Figure S6E,G). Collectively, collagen on the injured brain surface built a tight base to gather sufficient macrophages that required messenger nucleotide-mediated migration. They rapidly constructed a tightly connected honeycomb network structure to build a barrier that quickly sealed the brain surface and kept the tissue inside the brain. This process was essential for the efficient regeneration and eventual survival of the injured zebrafish larvae (Figure 8H).

## 3. Discussion

Macrophages are widely distributed and diverse types of immune cells. The canonical functions of macrophages in phagocytosis involving the secretion of trophic factors has been intensively investigated [62]. However, novel roles of macrophages are gaining increasing attention. In the present study, we constructed a standardized brain injury model and observed the rapid aggregation of macrophages and microglia on the injury surface to build a dense structure. Roth’s research showed that, after acute brain injury, the brain induced a purinergic receptor-dependent inflammatory response that played a neuroprotective role, characterized by meningeal neutrophil aggregation and microglial remodeling of damaged glial limiting cells [35]. In this study, the microglia were referred to as “honeycomb” microglia and “jellyfish” microglia morphologically. In our study, we also saw a significant reduction in branching jellyfish-like *coro1a*^+^ cells and the honeycomb structures formed by their close aggregation. Although we quoted Roth’s “honeycomb” to name the dense structure formed by the aggregation of macrophages, the focus of our research was different.

The pros and cons of microglial phagocytosis after brain injury have been debated. Herzog provided evidence that microglial debris clearance was neuroprotective after brain injury in vivo [53]. It was necessary to discuss the phagocytosis of macrophages/microglia in the honeycomb network structures. On the one hand, it should be emphasized that each macrophage in this structure acted as a phagocyte to clear apoptotic debris, but the distribution of early macrophages did not depend on the distribution of apoptotic signals. Our results indicate that macrophages tended to form a blocking structure on the wound surface early after injury. Herzog’s study focused on the effect of microglia on secondary nerve injury, while our study focused on the earliest neuron loss, so there were different views on microglia phagocytosis. We also demonstrated that phagocytosis played a limited role in preventing the loss of neural tissue in the brain from the perspective of gene expression levels and loss of function experiments. On the other hand, our study emphasized the role of the honeycomb network structures formed by macrophages/microglia as a whole, rather than focusing on the role of each cell itself. Having a sufficient number of macrophages was necessary to prevent neuronal loss after traumatic brain injury. Our study emphasized a novel role for macrophages in building physical barrier through rapid and efficient aggregation.

In the early stages of cell death, purines and pyrimidines are released from dying cells [63]. Under stressful conditions, such as ischemia, pressure, or swelling, hemichannels are the key molecules in the regulation of purinergic receptor activation to release ATP [64]. “Find me” signals from apoptotic cells may be released in a neuronal activity-dependent manner through pannexin hemichannels [43]. Pannexin-1 is a hemichannel associated with calcium ions and ATP release, which is widely expressed in the zebrafish brain [41]. The application of inhibitors (CBX and Pro) or hydrolases partially inhibited the aggregation of macrophages, supporting the results of the previous studies showing that ATP was indeed a “find me” signal [43]. However, other signals released from the pannexin-1 channel [63], such as calcium signals, may also serve as inducers of macrophage recruitment. The specific mechanisms underlying the macrophage-mediated regulation of calcium signaling were open for further investigation. In addition, the roles of additional molecules, including interleukins and tumor necrosis factor-α (TNF-α), and the underlying mechanisms behind their involvement in regulating chemotaxis of immune cells remain worthy of systematic investigation.

Type IV collagen is the only type of collagen present in the basement membrane of vertebrates, which is different from other collagens [26]. A characteristic feature of collagen IV is the presence of 21–26 interruptions in the collagenous Gly-Xaa-Yaa triple repeats [65,66]. These interruptions in the collagenous domain provide molecular flexibility for network formation, some of which serve as cell-binding and interchain crosslinking sites [66]. Once collagen Ⅳ is secreted into the ECM, it can form distinct networks through the self-association of triple-helical molecules and provide molecular scaffolds for other ECM components, such as laminin, perlecan and proteoglycans [66]. Macrophages selectively adhere to denatured type I collagen [67]. Few studies have focused on whether type IV collagen can induce macrophage migration. Studies have shown that, following acute tissue injury, macrophage-derived amphiregulin in the tissue induced TGF-β activation and pericytes to differentiate into collagen-producing myofibroblasts, resulting in rapid tissue vascular remodeling and wound healing [68]. However, the direct interaction between macrophages and collagen remains largely unexplored.

The present study indicated that both type Ⅳ collagen and laminin could induce macrophage aggregation. Laminin was another important component of the basement membrane [69] and also participated in the regulation of cell migration [70]. However, only the supplementation of collagen can alleviate the edema-like phenotypes, which may be related to the special network structure of type Ⅳ collagen. Multiple studies highlighted the important role of collagen IV in cell migration, survival, proliferation and differentiation [71,72]. It has been proposed that type Ⅳ collagen is a binding substrate for a large number of cells, including platelets and endothelial cells [73]. This study was the first to suggest that collagen can protect the brain by encouraging macrophages to bind together tightly after injury. However, how collagen binds recruited macrophages together remains an open question. Sixma’s study pointed out that α2β1 integrin plays an important role in the adhesion of platelets to collagens and in maintaining the hemostasis of the blood vessel wall during vascular injury [74]. Integrin α2β1 is a widely expressed type I collagen receptor that also mediates laminin-binding in certain cell types [75]. Thus, it is worth exploring whether the interaction between collagen and macrophages is mediated by integrin.

Another concern is the source of collagen in the wound. According to our experimental results, macrophages and collagen can be co-stained to a certain extent. Therefore, we believe that macrophages could express collagen after injury. However, we cannot distinguish the source of collagen due to the presence of a collagen layer within the skin in the basement membrane area. In addition, macrophages may also phagocytose collagen, which may lead to false positive identification of collagen production [76]. Fibroblasts were also considered to be a source of collagen IV due to the presence of various sources of fibroblast-like cells in healing wounds. However, fibroblasts can only produce low levels of collagen, so the current process of detecting fibroids using intracellular collagen is still complicated [77].

To summarize, our study revealed the important role of macrophages and collagen in forming a protective honeycomb network structure for the maintenance of brain tissue integrity after injury. This study provided a new insight into the pathogenesis of cerebral edema and may provide new evidence for the protective role of macrophages after TBI.

## 4. Materials and Methods

### 4.1. Zebrafish

AB, *Tg(apoe:GFP)* [78], *Tg(coro1a:DsRed)* [37], *Tg(coro1a:eGFP)*, *Tg(coro1a:Dendra2-NTR)*, *Tg(coro1a:Kaede)*, *Tg(HuC:GFP)* [32], *Tg(mpeg1:eGFP)* [36] and *Tg(krt4:Sce.Abp140-Venus)^cy22^* [79] strains were maintained according to the guidelines of experimental animal welfare from the Ministry of Science and Technology of the People’s Republic of China (2006). All animal experiments were performed under approval from the Institutional Review Board of Southwest University (Chongqing, China). Embryos were obtained after natural spawning and treated in E3 buffer with 0.003% PTU (1-phenyl-2-thiourea, Sigma-Aldrich, St. Louis, MO, USA) to prevent pigment formation.

### 4.2. Tg(coro1a:Dendra2-NTR) Transgenic Line Construction

To generate *Tg(coro1a:Dendra2-NTR)* transgenic lines, the *pTAL* vectors that contained *coro1a* regulatory elements were inserted by the Dendra2-NTR segment using restrictive endonuclease (Xma1/Sma1 and Cla1) and T4 ligase. The *coro1a:Dendra2-NTR* plasmid (30 pg) was mixed with Tol2 transposase mRNA (250 pg) and injected into one-cell embryos. The heritable *Tg(coro1a:Dendra2-NTR)* transgenic lines were identified based on the fluorescence of Dendra2^+^. Cell ablation in zebrafish larvae was carried out as previously described according to the MTZ/NTR working principle [80]. The 2.5 dpf *Tg(coro1a:Dendra2-NTR)* larvae were incubated in metronidazole (MTZ) solution (10 mM in PTU egg water, Sigma-Aldrich, USA) for 12 h.

### 4.3. Generation and Genotyping of Cebpα Mutant Lines

Mutation in *cebpα* was generated by using the CRISPR/Cas9 system. The guide RNA’s target sequence was 5′-GGACTAGGTACGGGCGTCGG-3′ in exon1, which was in vitro synthesized by the mMESSAGE mMACHINE T7 transcription kit (Thermo Fisher Scientific, USA). A mixture of Cas9 mRNA and gRNA according to the manufacturer’s instruction (NewEngland Biolabs, USA) was injected into fertilized embryos at one cell stage. The *cebpα* mutant (*cepbα^+46/+46^*) was obtained by DNA sequencing. For genotyping the *cebpα* mutant, PCR was performed with the oligonucleotides *cebpα*-forward primer (5′-CGCCTACATTGATCCGTCTG-3′) and *cebpα*-reverse primer (5′-CGTCTCCAGTTCTCGCGTGAG-3′).

### 4.4. Cell Line and Cell Culture

The HeLa, ND7/23, Raw264.7 cell lines were cultured following a standardized protocol. Briefly, cells were maintained at 37 °C with 5% CO_2_. The medium contained high-glucose DMEM with glutamine and sodium pyruvate (Biological industries, Israel), 10% inactivated fetal bovine serum (Biological industries, Israel), 1% l-glutamine (Beyotime, China), 1 µg ml^−1^ Mycoplasma Removal Agent Plus (Beyotime, China) and 1% penicillin-streptomycin-amphotericin B solution (Beyotime, China). The cells were grown in the cell incubator (Shanghai Yiheng Technology Co., Ltd., China) following a standard manual.

For the cell adhesion test, we coated 100 µL of the collagen IV protein solution (20 µg mL^−1^) or 0.002% glacial acetic acid on a 96-well plate at 4 °C overnight. Discarding the coat solution, we then washed with PBS three times before sealing with 1% BSA (200 µL well^−1^) at 37 °C for 1 h. After washing with PBS three times, 100 µL of serum-free medium was added to the cell suspension (the total number of cells per well was 1 × 10^4^). The cells were placed horizontally at 37 °C and cultured in a 5% CO_2_ cell incubator for 1 h and the results were photographed using the NIS-Elements BR (v.5.10.00) software. The same method was used to coat with 100 µL of the laminin protein solution (20 µg mL^−1^) and 100 µL of the BSA protein solution (20 µg mL^−1^). Collagen IV (20 µg mL^−1^) and laminin (20 µg mL^−1^) were decomposed by collagenase (20 µg mL^−1^) at 37 °C for 1 h. For the heat assay, collagen IV was denatured at 100 °C for 20 min.

The cell lines used in this study were obtained from the Cell Resource Center, Shanghai Institute of Life Sciences, Chinese Academy of Sciences.

### 4.5. Larval Zebrafish Traumatic Brain Injury

Larvae (3 dpf) were anaesthetized in 0.01% tricaine (MS-222, Sigma-Aldrich, USA). The glass needle used was 45.5 microns thick and kept at a horizontal angle of 45° while performing the stab. A puncture was performed at the injury center, resulting in a large area of neuronal damage. The width of the needle’s insertion was 130 microns and the depth was 150 to 180 microns. The center of the injury was in the left midbrain of zebrafish. After the stabbing, a lesion circle with a diameter of about 100 microns was formed. We repeated the needle stab exercises to make sure that we attained the same injury level. All of the statistical experiments on edema involved in this study were performed more than 5 times.

### 4.6. Measurement of Edema Volume

The volume of edema was analyzed using ImageJ (National Institutes of Health: NIH, USA). Each plane of the edema was segmented by image thresholding and the volume of the edema was calculated as the sum of the area of edema of each z slice multiplied by z spacing.

### 4.7. Whole-Mount In Situ Hybridization (WISH) and Neutral Red Staining

RNA probes were synthesized by DIG RNA Labeling kit (Roche, Basel, Swiss). WISH and NR staining (N6264, Sigma Aldrich, USA) were performed as previously described. To verify the authenticity of the experimental results, the WISH experiments and the NR staining were performed twice. Representative images were obtained using a Discovery.V20 microscope (Carl Zeiss, Jena, Germany).

### 4.8. Immunofluorescence Staining

The zebrafish larvae were fixed using 4% PFA at room temperature for 4 h. After dehydration in 30% sucrose in PBS at 4 ℃ for 1 day, the fixed larvae were embedded in OCT compound (Sakura, 4583, Japan). Immunofluorescence staining was performed on 10 µm cryosections. Sections were blocked with 2% fetal bovine serum (FBS) in phosphate-buffered saline (PBS) for 30 min to minimize unspecific binding. The samples were incubated overnight with primary antibodies at 4 ℃ and all primary antibodies were diluted with 5% FBS (1:400) prior to use. Subsequently, the samples were washed several times with PBST and incubated with secondary antibody (1:400 dilution) in 5% FBS at room temperature for 4 h. Nuclei were visualized by staining with DAPI (Roche, 10236276001) 1:1000 in PBS for 30 min. The images were taken with an LSM700 or LSM880 confocal microscope. Antibody and reagent information are provided in Table S1.

### 4.9. TUNEL Assay and AO Staining

TUNEL assay and acridine orange (AO) staining are commonly used to identify apoptotic cells in zebrafish embryos [81]; however, TUNEL only detects fixed embryos, while AO staining has the advantage of rapidly analyzing apoptosis in live embryos. The TUNEL (In Situ Cell Death Detection Kit and TMR Red Kit, Roche, Swiss) assay was performed according to the manufacturer’s instructions. For AO staining, larval zebrafish were incubated with 2 µg ml^−1^ acridine orange for 40 min. The images were taken with an LSM700 confocal microscope. The positive signals of TUNEL^+^ and AO^+^ were counted using ImageJ.

### 4.10. Edu Assay

For the Edu incorporation assay, the Click-iTEdU Imaging Kit (Invitrogen, Waltham, MA, USA) was used. The injured zebrafish larvae could resume normal swimming 30 min after the stab wound. At this point, 1 nL of 10 mM Edu was injected into the pericardium of 3 dpf zebrafish larvae. The injected zebrafish were returned to the 28.5 °C incubator for 1.5 h and then fixed with 4% PFA. We followed the manufacturer’s instructions for subsequent Edu staining steps.

### 4.11. Lyso-Tracker and Mito-Tracker Assay

Larvae were incubated in MitoTracker^TM^ Deep Red (M22426, Thermo Fisher Scientific, USA) in egg water (1:500) for 40 min protected from light.

LysoTracker™Red DND-99 (L7528, Thermo Fisher Scientific, USA) was used (1:1000 dilution). Larvae were incubated in the dark for 1 h and then rinsed with egg water several times.

Images were taken with Zeiss LSM700 and Airyscan in an LSM880 confocal microscope. See Supplementary Table S1 for reagent information.

### 4.12. Transmission Electron Microscopy (TEM)

The transmission electron microscopy assay was conducted by the molecular medicine testing center of Chongqing Medical University. We prepared samples according to their instructions. Briefly, 3 dpf zebrafish larvae were fixed with 4% glutaraldehyde solution (25% glutaraldehyde in PBS) (G5882, Sigma-Aldrich, USA) for further experiments and photographs.

### 4.13. Microscopy and Image Analysis

Time-lapse imaging to track injury-responding macrophages was performed using a Zeiss LSM700 confocal with environmental control (28.5 ℃). Images of macrophage structure were captured using an LSM880 fast Airyscan confocal microscope. Images were analyzed by using ZEN 2012 SP1 (black edition), ZEN 2012 (blue edition) and Image J 1.53e. The 3D conformational analysis was performed by Imaris×64 (v.9.0.1). Kaede photoconversion was carried out using the bleaching tool with a 405 nm diode laser. Irradiation at 405 nm results in an increase in the 543 nm/488 nm fluorescence ratio of the fluorescent proteins.

### 4.14. Clodronate Liposomes Assay

The injection of clodronate liposomes followed previous descriptions. Clodronate liposomes (40337ES08/10, 40338ES08/10, Yeasen, Shanghai, China) were injected in the original solution into the blood circulation of 2.5 dpf zebrafish. Observations were performed after 14 h.

### 4.15. ATP Inhibitors and Apyrase Assay

We administered 100 mM ATP (adenosine 5′-triphosphate disodium salt solution) (A6559, Sigma-Aldrich) and 100 U mL^−1^ apyrase solution (apyrase from potatoes, A6535, Sigma-Aldrich) prior to acupuncture in the injury center. PBS was used as the control. In the ATP inhibitor assay, 2.5 dpf wild type larvae were incubated in carbenoxolone (CBX) solution (50 µm in PTU egg water) or 50 µm probenecid (Pro) for 12 h after injury and the fish were left in the inhibitor after injury for observation 3 h later.

### 4.16. Collagen and Collagenase Injection Assay

Collagen (C5533, Sigma-Aldrich, USA) was dissolved in 0.1 M acetic acid to make a 1% stock solution. Collagenase (C5138, Sigma-Aldrich, USA) was prepared as a 0.1% stock solution and then diluted to 0.01% with water. Laminin (L2020, Sigma-Aldrich, USA) was diluted to a concentration of 0.1%. These were then injected into the injury center one hour before stabbing.

### 4.17. L-SOP Assay

O-phospho-L-serine (S5137, Selleck, China) powder was diluted into a 150 mM stock solution with water and the stock solution was diluted to 300 µm for use. The 2.5 dpf larvae were incubated in L-SOP for 12 h to 3 dpf. See Table S1 for chemical information.

### 4.18. Fluorescence-Activated Cell Sorting, RNA-Sequencing and Analysis

The 3 dpf *Tg(coro1a:Kaede)* larvae were stabbed and 1 h later, these green *coro1a*-Kaede^+^ cells at the injured site were changed to red by the laser. The labeled cells were isolated by fluorescence-activated cell sorting (FACS). Live cells (based on red fluorescence) were sorted into pre-prepared centrifuge tubes containing 2.5 µL of cell-protective solution (Configured by Gene Denovo Biotechnology Co., Ltd., Guangzhou, China)). Immediately after sorting, cells were centrifuged and frozen at −80 ℃ before being sent to Gene Denovo for RNA-Seq.

### 4.19. Quantification and Statistical Analysis

In this study, each experiment was repeated at least once and measured at least three times. Then the measured data was averaged to get the final data to reduce measurement error. The number of macrophages and the signal counts for in situ hybridization experiments by analyzing particles in ImageJ 1.53e software. Quantitative data (mean ± SEM) were double confirmed and analyzed by GraphPad Prism 6.01 with Student’s unpaired two tailed *t*-test. Chi-square tests were performed using IBM SPSS Statistics software to analyze the relationship between macrophage loss and edema-like symptoms and the relationship between edema-like symptoms and mortality.

## Data Availability

The data that support the findings of this study are available from the corresponding authors upon request.

## Supplementary Materials

The supporting information can be downloaded at: https://www.mdpi.com/article/10.3390/ijms231810551/s1.
