# Supplementary material for: Macrophages Rapidly Seal off the Punctured Zebrafish Larval Brain through a Vital Honeycomb Network Structure
Source: Int J Mol Sci. 2022 Sep 11;23(18):10551. doi: 10.3390/ijms231810551 (PMC9503817; doi:10.3390/ijms231810551)
Supplement: Supplementary file 1 [file ijms-23-10551-s001.zip › ijms-1813322-supplementary.pdf]

Macrophages rapidly seal off the punctured zebrafish larval brain through a vital honeycomb network structure

Dandan Zou[1], Jie Qin[1], Wenlong Hu[1], Zongfang Wei[1], Yandong Zhan[1], Yuepeng He[1], Congjian Zhao[2] and Li Li[3*]

[1] Key Laboratory of Freshwater Fish Reproduction and Development, Ministry of Education,Institute of Developmental Biology and Regenerative Medicine, Southwest University, Beibei, Chongqing 400715, China

[2] Chongqing Engineering Research Center of Medical Electronics and Information Technology, School of Biomedical Engineering and informatics, Chongqing University of Posts and Telecommunications, Chongqing 40065, China

[3] Research Center of Stem Cells and Ageing, Chongqing Institute of Green and Intelligent Technology, Chinese Academy of Sciences, Chongqing 400714, China

[*]Correspondence : lili@cigit.ac.cn

Table S1. Summary of information on major antibodies and reagents.

REAGENT or RESOURCE	SOURCE	IDENTIFIER
**Antibodies**		
Mouse monoclonal anti-DsRed (E-8)	Santa Cruz Bio-technology	Cat# sc-390909 RRID: AB_2801575
Mouse monoclonal anti-HuC/HuD	Thermo Fisher Scientific	Cat# A-21271 RRID: AB_221448
Mouse Caspase 3 (5E1)	GeneTex	Cat# GTX34164 RRID: AB_2887734
Goat Anti-GFP	Abcam	Cat# Ab6658 RRID: AB_305631
Rabbit anti-Collagen IV	Abcam	Cat# ab6586 RRID: AB_305584
Rabbit anti-Laminin	Sigma-Aldrich	Cat# L9393 RRID: AB_477163
Rabbit polyclonal lgG anti-b-catenin	Sigma-Aldrich	Cat# 06-734 RRID: AB_310231
Rabbit anti-Integrin Linked ILK	Abcam	Cat# ab236455
Donkey polyclonal anti-Goat IgG (H+L) Highly Cross-Adsorbed Secondary Antibody, Alexa Fluor 488	Invitrogen	Cat# A-11055 RRID: AB_2534102
Donkey polyclonal anti-Mouse IgG (H+L) Highly Cross-Adsorbed Secondary Antibody, Alexa Fluor 555	Invitrogen	Cat# A-31570, RRID: AB_2536180
Donkey polyclonal anti-Rabbit IgG (H+L) Highly Cross-Adsorbed Secondary Antibody, Alexa Fluor 555	Invitrogen	Cat# A-31572, RRID: AB_162543
Donkey polyclonal anti-Rabbit IgG (H+L) Highly Cross-Adsorbed Secondary Antibody, Alexa Fluor 647	Invitrogen	Cat# A-31573, RRID: AB_2536183
Donkey polyclonal anti-Mouse IgG (H+L) Highly Cross-Adsorbed Secondary Antibody, Alexa Fluor 647	Invitrogen	Cat# A-31571, RRID: AB_162542
**Chemicals**		
Glutaraldehyde	Sigma-Aldrich	G5882
Tricaine	Sigma-Aldrich	MS-222

DAPI	Roche	10236276001
Metronidazole	Sigma-Aldrich	A600633-0025
Clodronate Liposomes	Yeasen	40337ES08/10
PBS Liposomes	Yeasen	40338ES08/10
Adenosine 5′-triphosphate disodium salt solution	Sigma-Aldrich	A6559
Apyrase	Sigma-Aldrich	A6535
Collagen	Sigma-Aldrich	C5533
Collagenase	Sigma-Aldrich	C5138
Laminin	Sigma-Aldrich	L2020
O-Phospho-L-serine	Selleck	S5137
Carbenoxolone disodium salt	Sigma-Aldrich	C4790
Probenecid (Benemid)	Selleck	S4022
**Critical commercial assays**		
In Situ Cell Death TMR	Roche	12156792910
Click-iT™ EdU Alexa Fluor™ 647 Imaging Kit	Invitrogen	C10340
MitoTracker™ Deep Red	Thermo Fisher Scientific	M22426
LysoTracker™Red DND-99	Thermo Fisher Scientific	L7528

Supplemental figures and figure legends

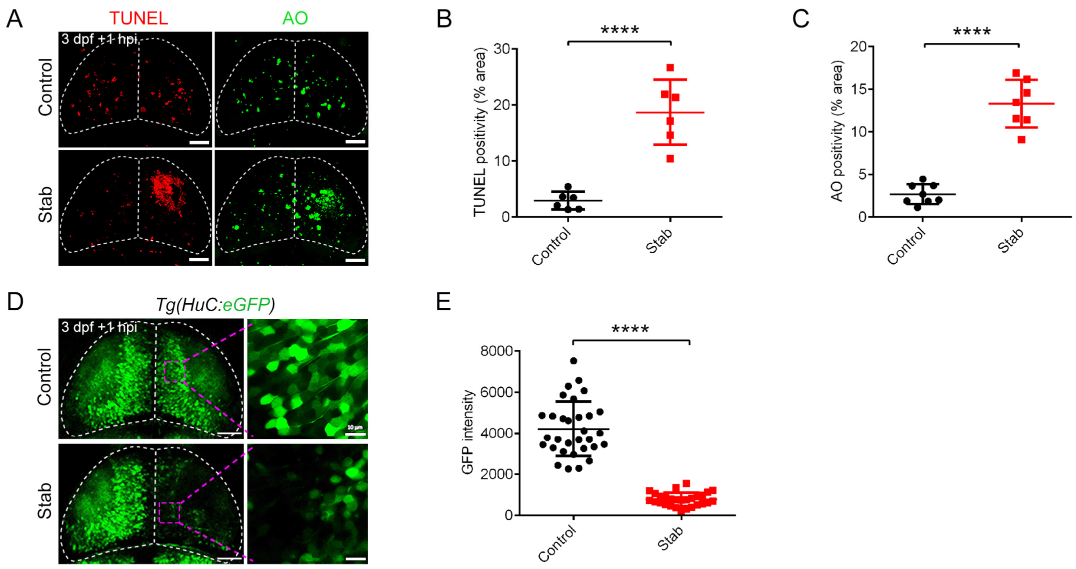

Figure S1. Our stab model can cause significant traumatic brain injury in zebrafish larvae. (A) TUNEL and AO staining of the injured site at 1 hpi. Scale bar, 50 μm. (B) Statistical analysis of $TUNEL^+$ signals. Control, 2.91 ± 0.65 % *n* =6; Stab, 18.69 ± 2.37 % *n* =6. (C) Statistical analysis of AO^+ signals. Control, 2.68 ± 0.41 % *n* =8; Stab, 13.32 ± 1.06 % *n* =7. (D) The fluorescence signal of *HuC*-GFP^+ after injury. The right panels are the magnifield box region in left. Scale bar, 50 μm (left), 10 μm (right). (E) Statistic analysis of the GFP intensity in (D). Control, 4225.00 ± 238.90 *n* =31; Stab, 777.70 ± 61.55 *n* =28. (Data are shown as mean ± SEM. ****, *p*<0.0001.)

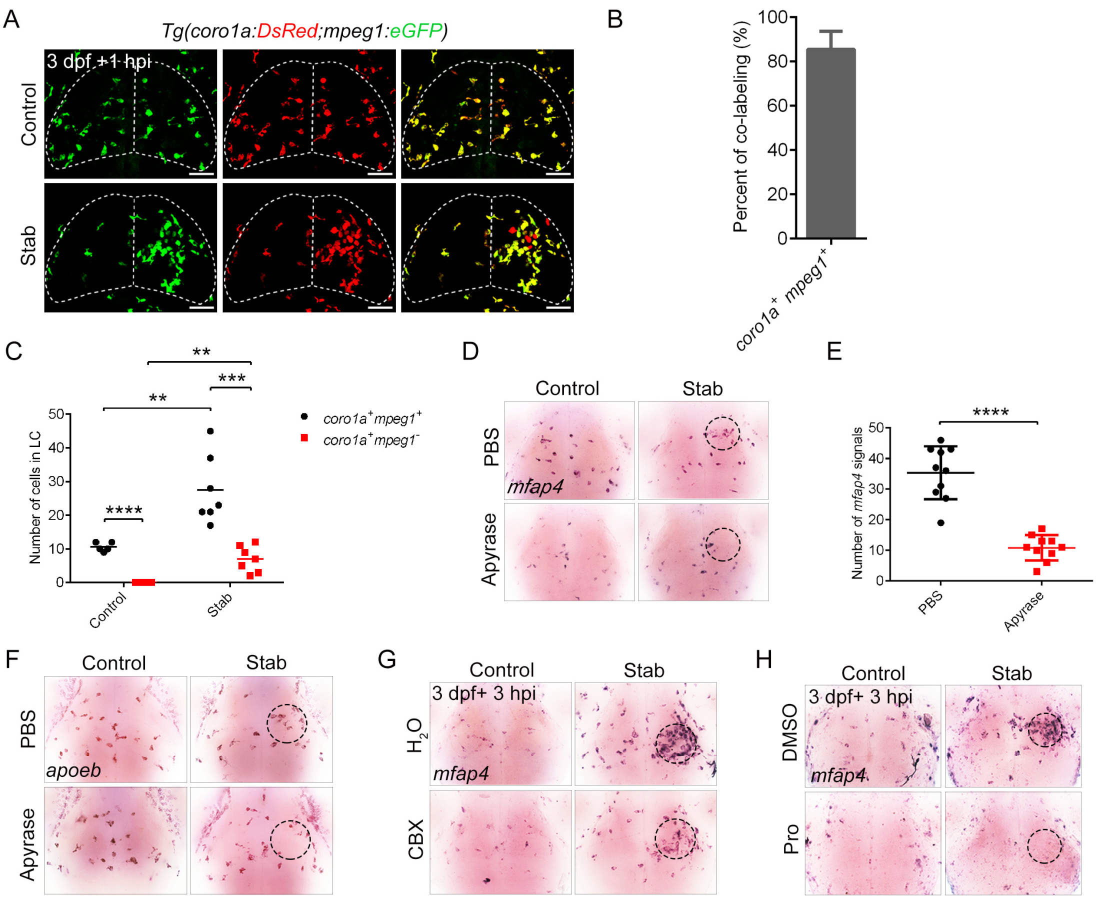

Figure S2. Recruitment of macrophages is mediated remotely by nucleotides. (A) The distribution of *coro1a*$^+$ and *mpeg1*$^+$ cells in *Tg(coro1a:DsRed;mpeg1:eGFP)* embryos. Scale bar, 50 µm. (B) Co-staining ratio of *coro1a*$^+$ and *mpeg1*$^+$ cells in lesion center after injury. 85.54 ± 2.330 % *n* =12. (C) Statistic analysis of the cell numbers of *coro1a*$^+$ *mpeg1*$^+$ (macrophages) and *coro1a*$^+$ *mpeg1*$^-$ (neutrophils or other cells) at injured site. Control, *coro1a*$^+$ *mpeg1*$^+$, 10.60 ± 0.60 *n* =5, *coro1a*$^+$ *mpeg1*$^-$, 0.00 ± 0.00 *n* =5; Stab, *coro1a*$^+$ *mpeg1*$^+$, 27.43 ± 3.82 *n*=7, *coro1a*$^+$ *mpeg1*$^-$, 7.00 ± 1.46 *n* =7. (D) WISH of *mfap4*. (E) Statistical analysis of *mfap4* signals after injury. PBS, 35.30 ± 2.73 *n* =10; Apyrase, 10.80 ± 1.31 *n* =10. (F-H) WISH of *apoeb* (F) and *mfap4* (G, H). (Data are shown as mean ± SEM. **, p<0.01; ***, p<0.001; ****, p<0.0001.)

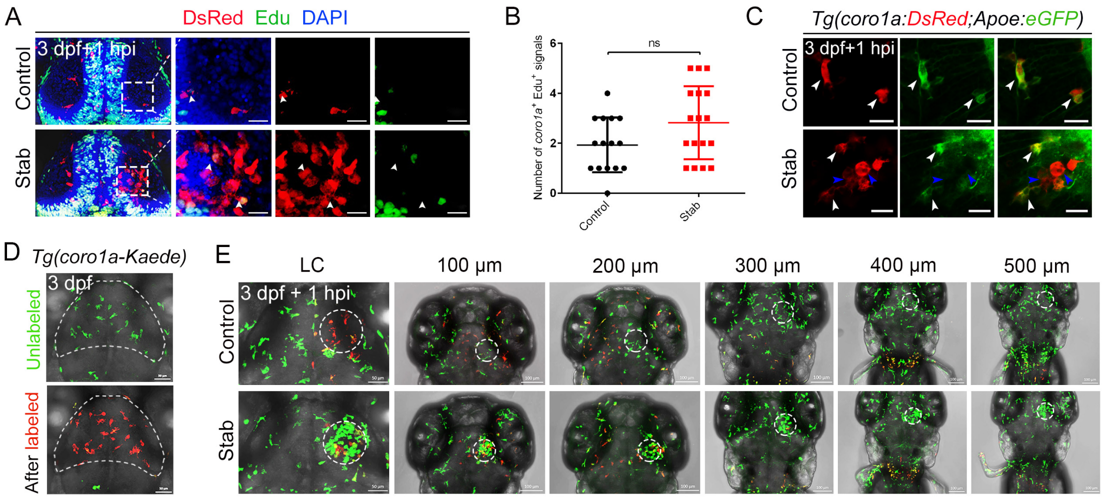

Figure S3. The source of aggregated macrophages. (A) Detection of proliferative *coro1a*-DsRed$^+$ cells by Edu. The white arrowheads indicate the DsRed$^+$Edu$^+$ signals. The right three panels are enlarged views of the boxed regions in the left panels. Scale bar, 50 μm (left), 20μm (right). (B) Statistic analysis of DsRed$^+$Edu$^+$ signals in (A). Control, 1.93 ± 0.28 n =15; Stab, 2.82 ± 0.36 n =17. (C) Aggregation of macrophages and microglia at 1 hpi in *Tg(coro1a:DsRed;apoe:eGFP)* embryos. Scale bar, 20 μm. (D) Macrophages in the midbrain and ventral brain change from green to red at 405 nm ultraviolet light in *Tg(coro1a:Kaede)* zebrafish. Scale bar, 50 μm. (D) *Coro1a*-Kaede$^+$ cells in different regions are labeled separately and their aggregated behaviors are imaged. Scale bar, 50 μm (LC), 100 μm (others). (Data are shown as mean ± SEM. *ns*, no significance.)

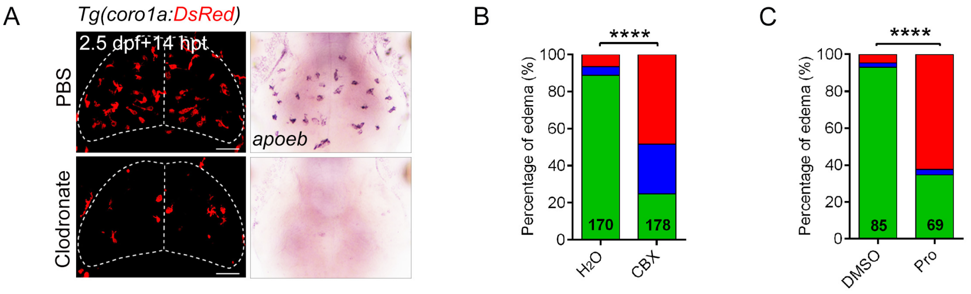

Figure S4. The absence of macrophages leads to the development of brain edema. (A) Detection of *coro1a*-DsRed$^+$ and *apoeb*$^+$ cells after injection of clodronate/PBS liposomes. Scale bar, 50 μm. (B, C) Statistics of edematous symptoms by treatment with carbenoxolone (CBX)/H_2O (B) and probenecid (Pro)/DMSO (C). (Data are shown as mean ± SEM. ****, p<0.0001.)

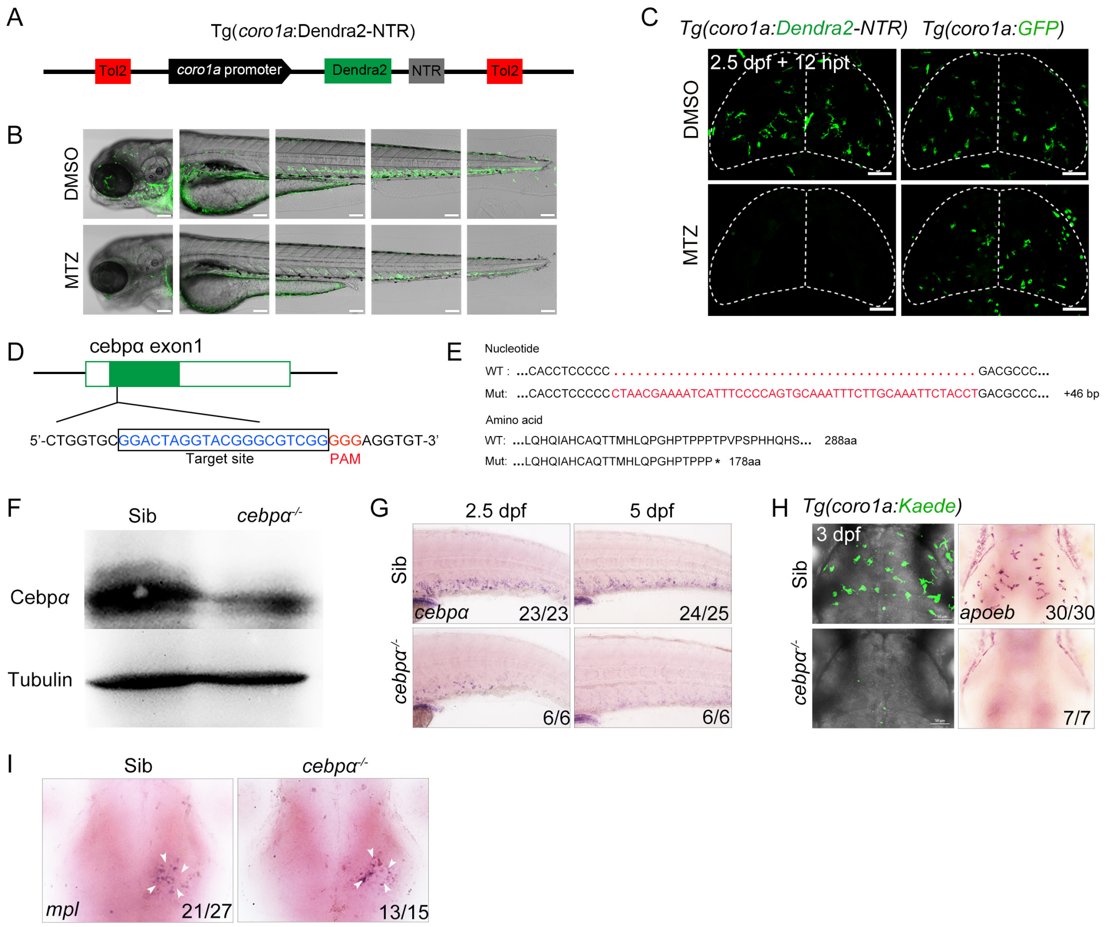

Figure S5. Specific loss of macrophages. (A) The schematic diagram of *Tg(coro1a:Dendra2-NTR)* construction. (B) The fluorescent images of *coro1a-Dendra2*$^{+}$ cells after treatment with DMSO/MTZ. Scale bar, 100 μm. (C) Phenotypes of macrophages treated with DMSO/MTZ in *Tg(coro1a:Dendra-NTR)* and *Tg(coro1a-GFP)* respectively. Scale bar, 50 μm. (D) Zebrafish *cebpα* locus shows gRNA target site in exon 1. (E) Nucleotide and amino acid sequences of *cebpα*$^{+46/+46}$. (F) Protein levels are analyzed by western blotting using an antibody against Cebpα. Tubulin, 55 kDa; Cebpα, 31 kDa. (G) WISH of *cebpα*. (H) The fluorescent and WISH images of Kaede^{+}, and *apoeb*$^{+}$ signals in sibling and *cebpα* mutant. Scale bar, 50 μm. (I) WISH of *mpl*.

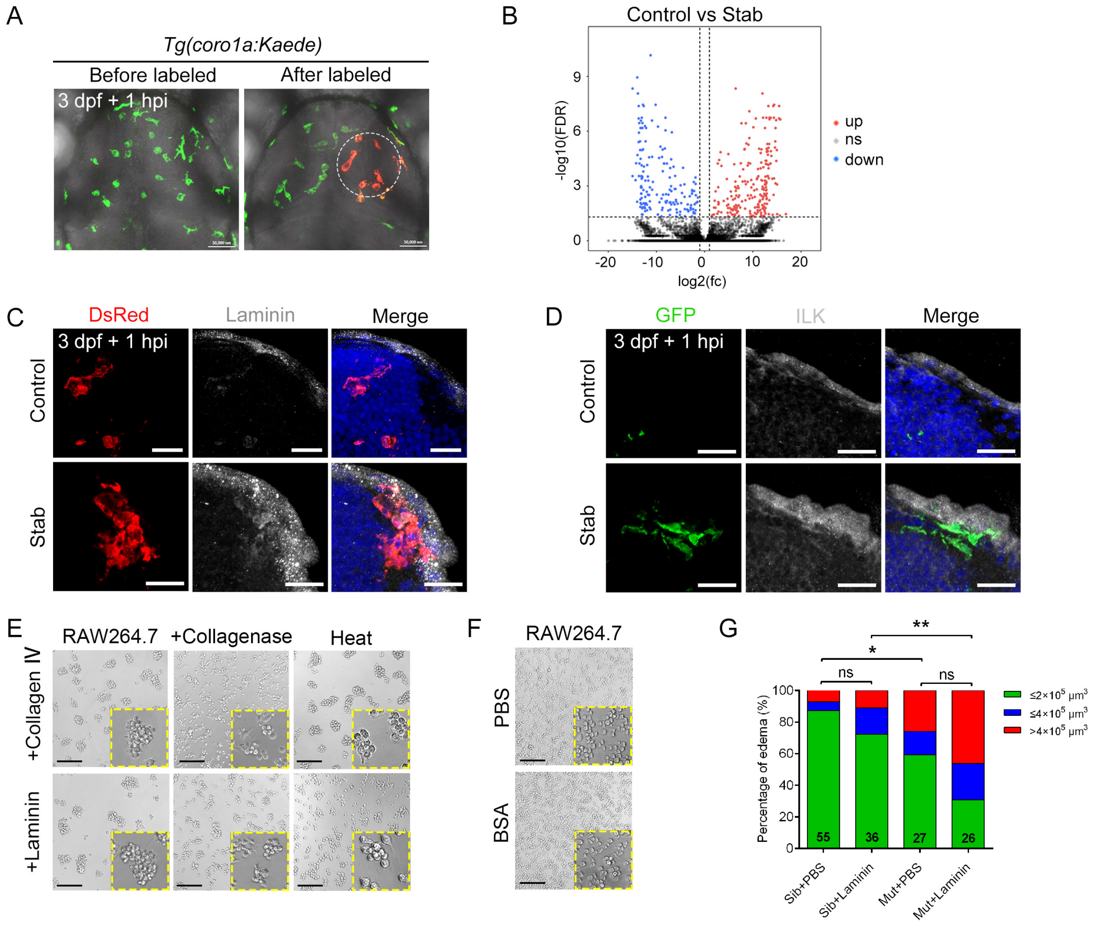

Figure S6. Collagen and laminin can induce aggregation of macrophages. (A) *Coro1a*-Kaede^{+} cells in the lesion center are labeled red and isolated for RNA-seq. Scale bar, 50 μm. (B) Volcano Plot of the transcriptomic comparison. (C) The immunofluorescence staining of DsRed and Laminin on the frozen sections of *Tg(coro1a:DsRed)* embryos at 1 hpi. Scale bar, 20 μm. (D) The immunofluorescence staining of GFP and integrin linked ILK on the frozen sections of *Tg(coro1a:eGFP)* embryos at 1 hpi. Scale bar, 20 μm. (E) Aggregation of RAW264.7 cells under different treatment conditions. The inserted panels indicate the magnified images. Scale bar, 100 μm. (F) Observation of RAW264.7 cells with BSA added. Scale bar, 100 μm. (G) Statistics of edematous symptoms with laminin/PBS injection in *cebpa* sibling and mutant after injury. (Data are shown as mean ± SEM. *ns*, no significance; *, $p<0.05$; **, $p<0.01$.)

Video Legends

Movie S1. Time-lapse live imaging on 3 dpf *Tg(coro1a:DsRed).*

Movie S2. The morphology and aggregation of macrophages under normal or stabbed (1 hpi) conditions at 3 dpf.

Movie S3. 3D distribution of macrophages and neurons in one side of zebrafish midbrain under normal or stabbed (1 hpi) conditions at 3 dpf.

Movie S4. Spatial distribution of macrophages and apoptotic signals (AO^{+}) under normal or stabbed (1 hpi) conditions at 3 dpf.
